# Assessing Sternal Dimensions for Sex Classification: Insights from a Greek Computed Tomography-Based Study

**DOI:** 10.3390/diagnostics15131649

**Published:** 2025-06-27

**Authors:** Konstantina Vatzia, Michail Fanariotis, Maciej Bugajski, Ioannis V. Fezoulidis, Maria Piagkou, Marianna Vlychou, George Triantafyllou, Ioannis Vezakis, George Botis, Stavroula Papadodima, George Matsopoulos, Katerina Vassiou

**Affiliations:** 1 Faculty of Medicine, University of Thessaly, Biopolis, 41110 Larissa, Greece; dr.vatzia@gmail.com; 2 Department of Radiology, Sykehuset Telemark HF, 3710 Skien Telemark, Norway; fanmih@gmail.com; 3 Maria Sklodowska-Curie Institute of Oncology, 31-125 Krakow, Poland; md.bugajski@gmail.com; 4 Department of Radiology, University Hospital of Larissa, Biopolis, 41110 Larissa, Greece; fezoulid@gmail.com (I.V.F.); mvlychou@med.uth.gr (M.V.); 5 Department of Anatomy, School of Medicine, Faculty of Health Sciences, National and Kapodistrian University of Athens, 11527 Goudi, Greece; georgerose406@gmail.com; 6 Biomedical Engineering Laboratory, School of Electrical and Computer Engineering, National Technical University of Athens, 15780 Athens, Greece; ivezakis@biomed.ntua.gr (I.V.); botis_g@biomed.ntua.gr (G.B.); gmatsopoulos@biomed.ntua.gr (G.M.); 7 Department of Forensic Medicine and Toxicology, School of Medicine, National and Kapodistrian University of Athens, 11527 Goudi, Greece; stpapd@gmail.com; 8 Department of Anatomy, Faculty of Medicine, University of Thessaly, Biopolis, 41110 Larissa, Greece; avassiou@gmail.com

**Keywords:** sternum, sex determination, morphometry, anatomy, forensic anthropology, computed tomography, variation

## Abstract

**Background/Objectives:** This study aimed to assess the potential of sternal morphometric parameters derived from multidetector computed tomography (MDCT) for sex estimation in a contemporary Greek population. A secondary objective was to develop and evaluate statistical and machine learning models based on these measurements for forensic identification. **Methods**: Sternal measurements were obtained from chest MDCT scans of 100 Greek adults (50 males, 50 females). Morphometric variables included total sternum length, surface area, angle, and index (SL, SSA, SA, and SI); manubrium length, width, thickness, and index (MBL, MBW, MBT, and MBI); sternal body length, width, thickness, and index (SBL, SBW, SBT, and SBI); and xiphoid process length and thickness (XPL and XPT). Logistic regression and a Random Forest classifier were applied to assess the predictive accuracy of these parameters. **Results**: Both models showed high classification performance. Logistic regression identified MBL and SBL as the most predictive variables, yielding 91% overall accuracy, with 92% sensitivity and 90% specificity. The Random Forest model achieved comparable results (91% accuracy, 88% sensitivity, 93% specificity), ranking SSA as the most influential feature. **Conclusions**: MDCT-derived sternal morphometry provides a reliable, non-invasive method for sex estimation. Parameters such as MBL, SBL, and SSA demonstrate strong discriminatory power and support the development of population-specific standards for forensic applications.

## 1. Introduction

The term “sternum” originates from the Greek “sterno”, meaning “chest.” It is a long, flat bone located in the midline of the anterior thoracic wall, composed cranio-caudally of the manubrium (MB), sternal body, and xiphoid process (XP) [1,2,3]. Anatomically, it articulates with the clavicles and costal cartilages of the first to seventh ribs and forms key joints, such as the manubriosternal joint (MBSJ) proximally and the sternoxiphoid joint (SXJ) [4,5] distally [2,6,7,8], where the sternal angle (SA) forms [2,6,7,8]. XP exhibits a high degree of anatomical variation [9].

Developmentally, the sternum originates from two mesenchymal bars that fuse at the midline by the end of gestation. Ossification commences in the ninth month of development, forming sternebrae (SBr), which subsequently fuse by the age of twenty-five. The mesenchymal bars converge craniocaudally at the midline, resulting in the cartilaginous sternum. The MB and sternum body (SB) ossification centers merge before birth, and the XP calcifies by the age of six years. Any failure in the fusion process may lead to congenital anomalies of the sternum [1,3,5,10,11]. However, specific information regarding the ages at which the development of the sternum is completed remains limited [12].

Sex estimation plays a central role in forensic identification, especially in fragmented or decomposed remains, where complete skeletal recovery is not possible [13,14]. Bone measurements are essential in sex estimation and play a critical role in resolving medicolegal cases [1,11]. While the pelvis and skull are the most frequently studied and diagnostically reliable elements [2], other structures, such as the sternum and, to a lesser extent, the orbital region, have received comparatively limited attention in the literature [15,16,17,18,19,20].

Advances in imaging, particularly multidetector computed tomography (MDCT), now allow for detailed, noninvasive assessment of sternal morphology. Prior research has demonstrated that three-dimensional (3D) sternal metrics—including length, width, and surface area—can distinguish sex with moderate to high accuracy (55.6–93.7%). Discriminant analysis of bone combinations achieved 94.7% accuracy in sex determination by measuring the ribs, sternum, and vertebrae [21,22,23,24,25]. However, expressions of sexual dimorphism vary across populations, emphasizing the need for population-specific standards in osteometric analysis [7].

The sternum holds forensic value due to its high recovery rate in decomposed or fragmented remains, making it a practical candidate for sex and age estimation [21]. Multiple studies have demonstrated its diagnostic utility in osteometric sex assessment [1,2,3,4,5,6,7,8,11,22,23,24,25]. Statistical methods applied to sternal morphometry include proportions, limiting points, and discriminant function analysis. However, the accuracy of sex estimation using sternal measurements varies across populations [2,6,23,24]. Given the population-specific expression of sexual dimorphism, such studies must be grounded in localized data to ensure forensic reliability [7].

This study investigates sternal morphometric parameters in a contemporary Greek population using MDCT. The primary objective is to assess the diagnostic accuracy of these features in sex estimation. A secondary aim is to develop and evaluate classification models—both statistical and machine learning-based—that may aid forensic anthropologists in population-specific sex identification. A terminological note: throughout this study, *sex* denotes biological distinctions, under forensic anthropological standards.

## 2. Materials and Methods

### 2.1. Patient Population

This retrospective MDCT study was conducted at the General University Hospital of Larissa, Greece, over six months (November 2022 to April 2023). The sample included 100 adult patients—50 males (61.38 ± 13.78 years) and 50 females (58.1 ± 17.7 years)—who underwent chest imaging for various clinical indications.

Inclusion Criteria: Patients were randomly selected to achieve balanced representation across sex and age ranges. All had complete medical records available during the MDCT examination. Imaging indications included evaluation of thoracic tumors (primary or metastatic), assessment of vascular or airway abnormalities, follow-up of known pulmonary lesions, and investigation of lung opacities observed on chest radiograph.

Exclusion Criteria: Patients were excluded if they presented with congenital or acquired sternal deformities, a history of thoracic trauma involving sternal fractures, prior sternal surgery, or pathological conditions affecting the sternum (e.g., masses, infections, infiltrations).

Note on Morphological Variants of the Sternum: Morphological sternal variants, such as bifid XPs or sternal foramina (SF), were included in the primary morphometric analysis unless they met exclusion criteria related to trauma, surgical alteration, or disease. These variants were also documented and evaluated separately as a secondary component of the study.

### 2.2. Multidetector Computed Tomography (MDCT) Protocol

All scans were performed using a 128-slice MDCT scanner (Philips Ingenuity CT 128, Amsterdam, The Netherlands). Patients were positioned supine, and imaging was conducted during the arterial phase, approximately 25 s after intravenous contrast administration. The scan field extended from the thoracic inlet to the adrenal glands, ensuring complete sternum coverage, including the body and XP of the sternum. Scanning parameters were detector collimation of 16 mm × 1.0 mm, tube voltage of 120 kVp, tube current of 200–400 mA with automated modulation, pitch of 1.0, slice thickness of 1 mm, and image matrix of 512 × 512. For contrast enhancement, 70 mL of non-ionic iodinated contrast medium—either iopromide (Ultravist, Bayer) or iodixanol (Visipaque, GE Healthcare)—was administered via the antecubital vein using a power injector at a rate of 3 mL/s. A 10 mL saline flush followed the contrast to optimize vascular opacification.

### 2.3. Image Post-Processing and Analysis

All MDCT datasets were post-processed using a dedicated workstation (IntelliSpace Portal, version 7.0.1.20482; Philips Medical Systems, Amsterdam, The Netherlands). An initial image review was performed on axial slices reconstructed at a thickness of 1 mm. Multiplanar reconstructions (MPR) in the sagittal and coronal planes were generated using a bone algorithm to optimize visualization of cortical and trabecular structures. To enhance anatomical assessment, 3D reconstructions (3DR) were created using both Maximum Intensity Projection (MIP) and Volume Rendering Technique (VRT). Due to the natural angulation at MBSJ and SXJ, coronal curved MPR was employed to depict the entire SL in a continuous plane. Two radiologists with 4 and 25 years of experience in thoracic imaging performed image analysis independently. Final measurements and interpretations were reached by consensus to ensure diagnostic consistency.

### 2.4. Sternum Measurements and Morphological Variants

**Figure 1:**
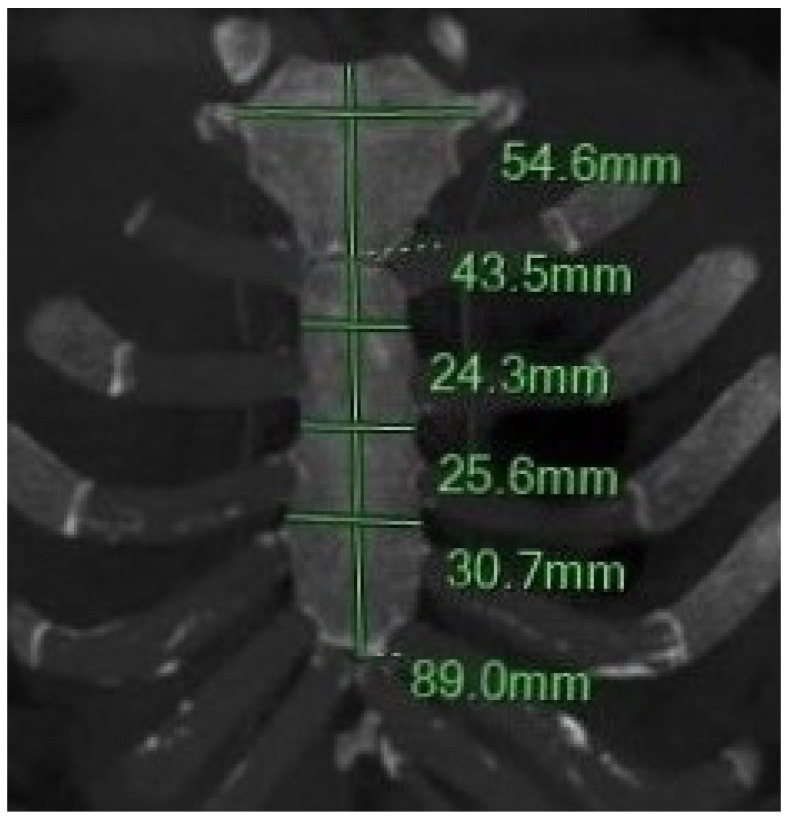
Coronal MIP MPR images illustrate the six measurements of the sternum: the length and width of the manubrium and sternal body, as well as the sternal body width at the 1st, 2nd, and 3rd sternebrae.

**Figure 2:**
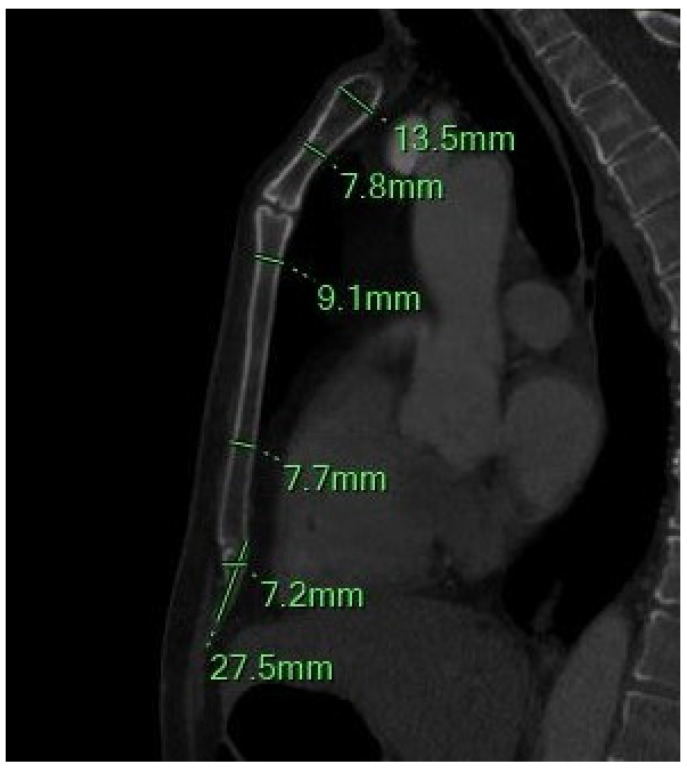
The sagittal MIP MPR image shows the minimum and maximum thickness of the manubrium, the thickness of the sternal body between the 2–3 and 4–5 sternebrae, and the length and width of the xiphoid process.

**Figure 3:**
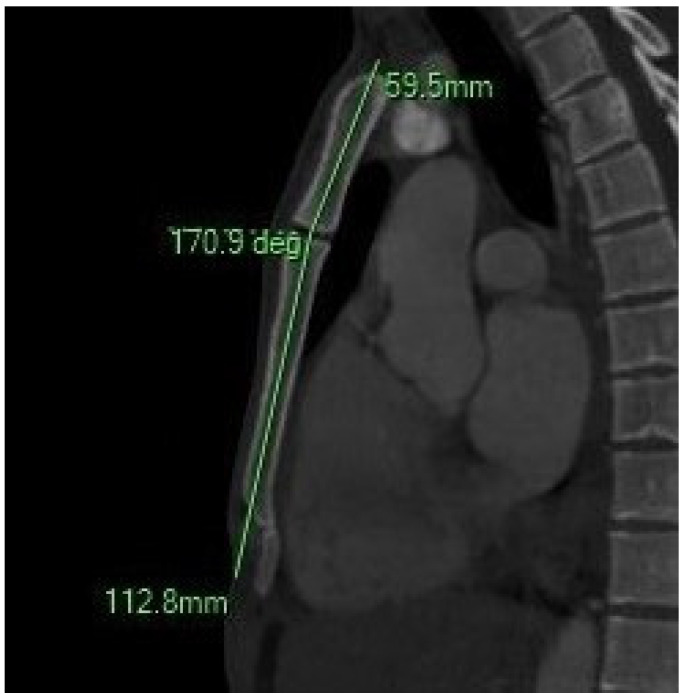
The sagittal MIP MPR image illustrates the sternal angle.

Sternal morphometric parameters were measured using reconstructed MDCT images, following established protocols from previous anatomical and forensic studies [1,2,3,4,5,6,7,8,9,10,11,15,16,17,18,19,21,22,23,24,25,26,27,28,29,30,31,32,33,34,35,36,37,38,39,40,41,42,43,44,45]. The following variables were assessed (Figure 1, Figure 2 and Figure 3):

#### 2.4.1. Manubrium (MB) Measurements

- MB length (MBL): Longest linear distance from the midpoint of the jugular notch (JN) to the MBSJ.
- MB width (MBW): Transverse distance between the midpoints of the first costal notches on the right and left.
- MB maximum Thickness (MBT_max_): Maximum anteroposterior thickness at the central point of the clavicular notch, measured perpendicular to the central longitudinal axis.
- MB minimum Thickness (MBT_min_): Minimum anteroposterior thickness at the midportion of the MB along the central longitudinal line.

#### 2.4.2. Sternum Body (SB) Measurements

- SB Length (SBL): Longest linear distance from the MBSJ to the SXJ.
- SB Width (SBW): Transverse distances between right and left costal notches at the 2nd intercostal space, 3rd, and 4th intercostal spaces (SBWa, SBWb, and SBWc).
- SB Thickness (SBT): Measured at the junctions of the 2nd–3rd and 4th–5th SBr, representing the anteroposterior depth.

#### 2.4.3. Xiphoid Process (XP) Measurements

- XP Length (XPL): Linear distance from the SXJ to the distal tip of the XP.
- XP Thickness (XPT): Maximum thickness measured between the SXJ and the XP’s distal endpoint.

#### 2.4.4. Combined and Index-Based Parameters

- Sternum Total Length (SL): Sum of the manubrium and body lengths: SL = MBL + SBL.
- Sternal Angle (SA): Formed between the following:
  1. A line parallel to the body and intersecting the midpoint of the JN.
  2. A second line parallel to the body and intersecting its midpoint.

#### 2.4.5. Indices

- Sternal Index (SI): (MBL/SBL) × 100 Reflects the proportion of manubrium length to sternum body length.
- MB Index (MBI): (MBW/MBL) × 100 Reflects the transverse-to-longitudinal ratio of the manubrium.
- SB Index (SBI): (SBWa/SBWc) × 100. Assesses the tapering pattern of the sternum body width.
- SSA (mm^2^): The estimated surface area of the sternum is calculated as ((MBL + SBL) × (MBW + SBWa + SBWc)/3)

Sternal morphological variants, such as bifid XP and SF, were included in the morphometric analysis unless they met exclusion criteria related to traumatic deformity, surgical alteration, or pathological involvement. These variants were evaluated as a separate and secondary analysis, independent of the primary morphometric dataset. Throughout this study, the term *sex* is used strictly to refer to biological and anatomical differences between males and females, under the standards of forensic anthropology and biological profiling. The term *gender* is deliberately avoided to maintain scientific precision.

### 2.5. Statistical Analysis

Basic descriptive statistics were calculated for all sternal measurements, including mean, standard deviation (SD), minimum, and maximum. Normality of data distribution was assessed using the Shapiro–Wilk test and visual inspection of Q–Q plots, confirming the appropriateness of parametric testing for most variables. Sex-related differences were evaluated using the Student’s *t*-test, with statistical significance at *p* < 0.001.

To assess the diagnostic performance of individual variables in sex estimation, the Receiver Operating Characteristic (ROC) curve analysis was performed. Optimal cutoff values for significant parameters were determined using Youden’s J index, which maximizes the sum of sensitivity and specificity. For each discriminative metric, sensitivity, specificity, positive predictive value (PPV), negative predictive value (NPV), and area under the curve (AUC) were reported.

A binary logistic regression model was constructed to predict sex based on selected morphometric features. MBL and SBL were included in the final model due to their strong statistical significance (lowest *p*-values), anatomical clarity, and interpretability. Although sternal surface area (SSA) exhibited the highest discriminative value in the Random Forest analysis, it was excluded from the logistic regression due to its composite nature and potential collinearity with primary variables. Preference was given to anatomically distinct and statistically stable predictors to enhance model robustness and clarity.

Although a formal a priori power analysis was not conducted, the consistently large effect sizes and highly significant differences (*p* < 0.001) across most parameters support the adequacy of the sample size for exploratory modeling and reliable pattern detection.

All statistical analyses were conducted using R statistical software (version 4.0) and Jamovi (version 2.1).

## 3. Results

### 3.1. Descriptive Morphometry and Statistical Significance

Sternal morphometric measurements for both sexes are summarized in Table 1. Statistical analysis revealed that all parameters were significantly different between males and females (*p* < 0.001), except for SA, SBT between the 2nd–3rd and 4th–5th sternebrae (2–3 and 4–5), and XPT. All derived indices are SI, MBI, SBI, and SA.

**Figure 4:**
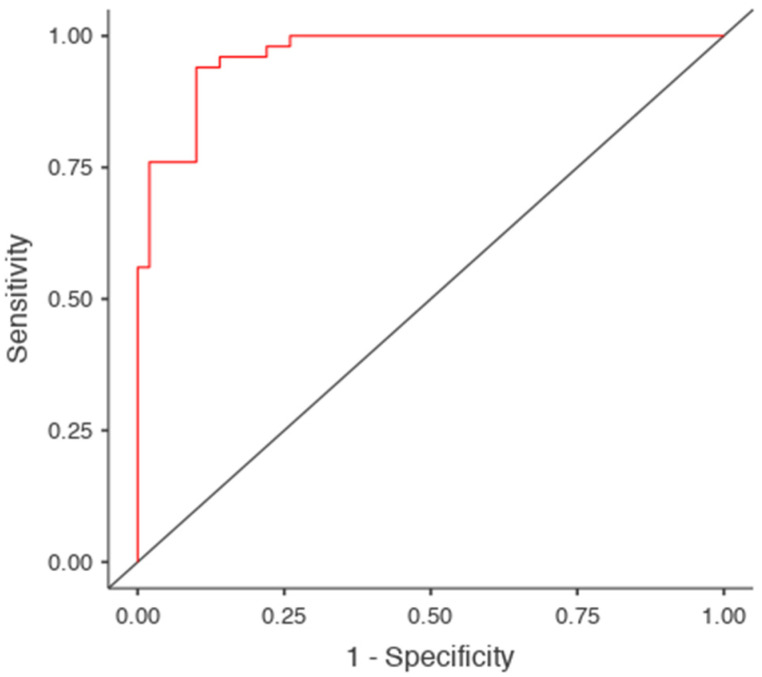
ROC curve for the logistic regression model using Manubrium Length (MBL) and Sternal Body Length (SBL) as predictors. The model achieved an area under the curve (AUC) of 0.97, indicating excellent discrimination between male and female subjects. The red line represents the model’s sensitivity–specificity tradeoff, while the diagonal grey line indicates random classification performance (AUC = 0.5).

As shown in Table 2, both predictors were highly significant and contributed independently to the model. The model correctly classified 92% of males (46/50) and 90% of females (45/50), yielding 91% accuracy, 92% sensitivity, 90% specificity, and 0.97 AUC (Figure 4).

ROC analysis was conducted to evaluate the discriminative performance of individual measurements. Figure 4 displays the ROC curve for the logistic regression model, which yielded an AUC of 0.97, indicating excellent classification performance. Optimal threshold values and AUCs for key individual predictors (based on Youden’s J): MBW: Cut-off = 70.9 mm, AUC = 0.847, SBL: Cut-off = 94.4 mm, AUC = 0.918, SL (MBL + SBL): Cut-off = 143.2 mm, AUC = 0.965, and SSA: Cut-off = 6039.5 mm^2^, AUC = 0.978 (Table 3).

**Figure 5:**
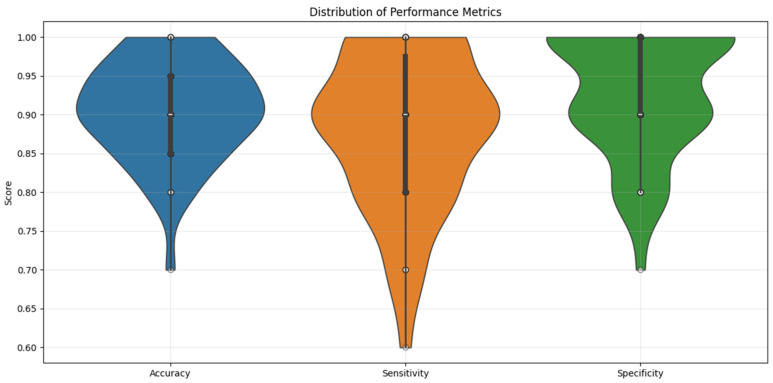
Violin plot illustrating the Random Forest Classifier’s performance metrics distribution across the ten repetitions of five-fold cross-validation for sex prediction.

Machine Learning Results—Random Forest Classifier. The Random Forest classifier achieved strong predictive performance across 50 iterations of stratified five-fold cross-validation: mean accuracy: 91% (±12.7), mean sensitivity: 88% (±19.5), and mean specificity: 93% (±15.6). Figure 5 illustrates the distribution of these performance metrics across all validation folds using violin plots. Analyzing feature importance based on the Gini index revealed the following top predictors: SSA: 0.3364, SBL: 0.1627, MBW: 0.1206, and MBL: 0.0593. These results reflect a key methodological contrast: while logistic regression emphasized linear, anatomically distinct predictors like MBL, the Random Forest model highlighted SSA—a composite metric—as the most influential feature, likely due to its capacity to capture complex, non-linear interrelations among variables. The least informative predictors included age: 0.0106, XPT: 0.0103, and SA: 0.0100.

**Figure 6:**
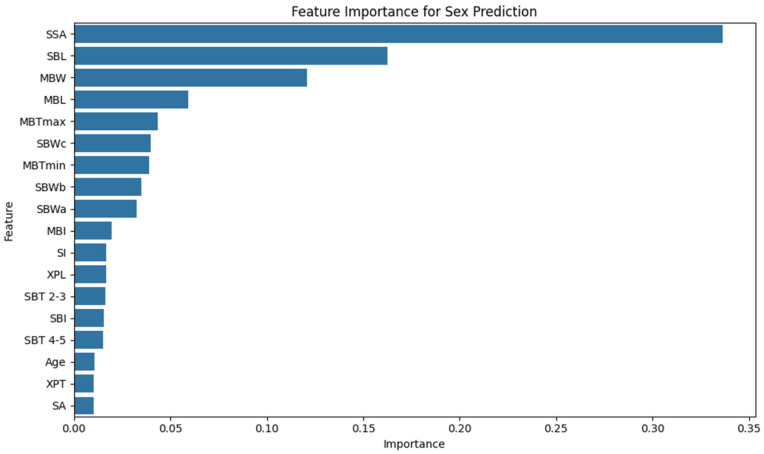
Feature importance plot for sex prediction. The bar chart illustrates the relative contributions of the biometric features to the Random Forest Classifier. SSA—sternum surface area, SBL—sternal body length, and MBW—manubrium width received the highest importance scores, indicating that these features significantly influence classification accuracy. Less influential variables included Age, XPT (xiphoid process thickness), and SA (sternal angle).

Figure 6 visualizes the relative importance of all features included in the model. To confirm the robustness of these rankings, permutation importance analysis was conducted. It produced a high concordance with Gini-based importance (Pearson r = 0.919). While values were more conservative, the same features ranked highest, with SSA again leading (Permutation: 0.2120 ± 0.034). The top five features—SSA, SBL, MBL, MBTmax, and MBW—remained consistent across both approaches.

**Figure 7:**
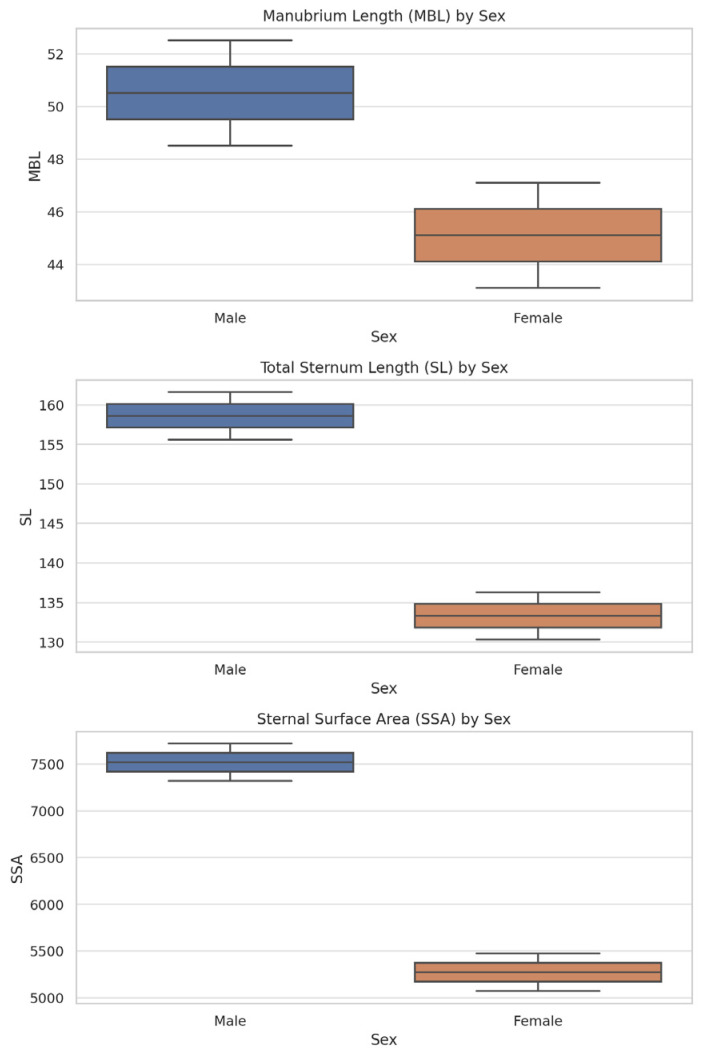
Boxplots for manubrium length (MBL), total sternum length (SL), and sternal surface area (SSA) by sex. All three parameters show significant differences (*p* < 0.001), supporting their diagnostic relevance.

Figure 7 presents box plots for MBL, SL, and SSA by sex to illustrate sex-based variability in the most relevant morphometric parameters.

### 3.2. Sternal Morphological Variants

A range of anatomical variants was observed in the study cohort and is summarized in Table 4. A double-ended XP in 44% of cases was the most frequently documented variation. Other common findings included ligamentous calcification (36%), xiphoidal foramen (30%), and sternal notch (30%). The rarest anomaly was a sternal cleft, observed in only one individual (1%). Sternal morphological variants were distributed relatively evenly across sexes, with no significant differences observed in individual variant frequencies (Fisher’s exact test, *p* > 0.05 for all). The double-ended XP was the most common variant in both males and females. While some features, such as elongated XP, appeared more frequently in males, the differences were insignificant in this sample. These findings suggest that although sternal variants are prevalent, they do not display strong sexual dimorphism and thus may have limited utility in sex estimation.

## 4. Discussion

One of the most critical components in constructing an adult’s biological profile from skeletal remains is the accurate sex estimation. Traditionally, forensic anthropologists have relied on morphological and metric analyses of the most sexually dimorphic and reliable skeletal elements for this purpose [6,46]. However, in cases where the pelvis or skull is missing or damaged, alternative skeletal elements like the sternum become valuable for sex estimation [6,8]. This limitation has led to growing interest in the sternum as an alternative site for sex and age estimation, owing to its relatively robust structure, frequent preservation, and emerging diagnostic value in morphometric analyses [47]. Artificial intelligence is also used in forensic sciences to estimate biological profiles [48,49].

### 4.1. Morphometric Details of the Sternum and Sex Determination Among Studies

One of the primary objectives in forensic anthropology is to accurately estimate sex from skeletal remains, a process vital to constructing a biological profile. While the pelvis and skull are traditionally considered the most reliable elements for sex estimation due to their pronounced sexual dimorphism [6,49,50,51]. These bones may be unavailable or fragmented in many forensic scenarios. In such cases, the sternum—owing to its durability and preservation—has emerged as a valuable alternative, particularly for sex and age estimation [6,8]. SL has historically been a point of investigation. Wenzel was the first to report a noticeable difference in SL between the sexes [28], focusing on the MBL/SBL ratio. Subsequently, Dwight [34] and Hyrtl [37] identified a recurring pattern: in females, the MBL approximates half the SBL, while in males, the SBL is at least twice the MBL. This led to the formulation of Hyrtl’s Law, which remains a foundational principle in sternal morphometrics. Ashley [28] further substantiated this with European data, finding that the male sternum was, on average, 18.2 mm longer than the female sternum (156.9 mm vs. 138.7 mm). Several population-specific threshold rules based on SL have since been proposed:

- Ashley’s “149 rule” for Europeans [28].
- Jit’s “136 rule” for North Indians [3].
- Dahiphale’s “129 rule” for the Marathwada region [32].
- Hunnargi’s “131 rule” for West India [2].
- Atesoglu’s “144 rule” for South-Eastern Anatolia [29]. However, absolute length-based thresholds may be unreliable across or even within populations due to secular trends and age-related variation in sternal growth [28,40]. Thus, morphometric values must be interpreted in a population-specific context. Based on our findings, a “143 mm rule” could be proposed for the Greek population, wherein an SL above 143 mm suggests male classification. This aligns with population-specific rules previously suggested by Ashley, Jit, and others, and could assist rapid field estimation where only sternal measurements are available. Our study confirms that the mean MBL and SBL in the Greek population align well with previous findings:
- Male MBLs (~50.5 mm) were consistent with data from Japanese [25], Spanish [36], and Turkish [29] populations.
- Female MBLs (~45.1 mm) aligned with values reported by Fernandez [22], Macaluso and Lucena [24], and Franklin [23].
- SBLs for males (~108.1 mm) and females (~89 mm) also matched findings from Croatian [43], Spanish [24], Turkish [11], and American [6] cohorts.

The differences between sexes in our Greek sample were notable: MBL: 5.37 mm, SBL: 19.95 mm, and SL (MBL + SBL): 25.32 mm. These differences suggest evident sexual dimorphism, with SL being a reliable predictor. For both sexes, MBL and SBL values in the Greek sample were comparable to those reported in Indian, Turkish, and Japanese populations, though Greek males tended to show slightly higher values. Notably, populations from India and the Middle East tend to have shorter sterna [52,53], while the highest measurements are reported among Croatians, followed by Greeks and Turks [25]. In our logistic regression model, the SL achieved a prediction accuracy of 91%, with the MBL emerging as the strongest individual predictor. However, our Random Forest machine learning model provided further insight: BL was more predictive than MBL (importance scores 0.16 vs. 0.06), highlighting the potential non-linear interactions among sternal parameter relationships more effectively captured by machine learning than traditional regression. The SSA was the most informative feature in the Random Forest model, a finding that supports Macaluso’s work [24], where SSA alone predicted sex with ~90% accuracy. Likewise, our study confirmed that MBW, with an AUC of 0.847, is also a strong predictor and may be especially valuable when the MB is the only preserved portion of the sternum. Conversely, some metrics, including SA, XPT, and calculated indices (SI, MBI, and SBI), were insignificant, confirming previous critiques of their low discriminative power [10,25,26]. Our findings and those from the literature support sternal measurements—particularly SL, SBL, MBL, MBW, and SSA—as reliable sex predictors, especially in cases where more commonly used bones are unavailable or fragmented [54,55]. Autopsies often require determining the sex of unidentified corpses, particularly in cases of decomposition, mutilation, or mass disasters [56]. Sternum measurements can be used in establishing the sex of unidentified corpses where the body is in advanced stages of putrefaction or a mutilated condition [57]. Nevertheless, sex estimation models are tailored to particular populations and cannot be generalized due to genetic and environmental variabilities [58]. Moreover, our study reinforces the importance of region-specific reference standards, given the variability introduced by population differences, age, and body habits.

### 4.2. Sternum Morphological Variants

In addition to morphometric analysis, the present study documented a variety of sternal morphological variants, several of which bear essential clinical and diagnostic implications. These variants are typically detected incidentally in living individuals through cross-sectional imaging modalities such as MDCT and should be recognized by radiologists to avoid potential misdiagnosis or unnecessary concern [9]. In our study, the most frequently observed variant was the double-ended XP, identified in 44% of cases. This is consistent with findings from a retrospective MDCT study of 1150 Greek patients, which reported sternal variants in 74.1% of cases [9]. In that population: Double-ended XP was also the most common variant (36.9%), followed by the single xiphoidal foramen (25.8%), sternal sclerotic band (12.8%), sternal notch (10.1%), xiphoidal ligament calcification (8.3%), SF (4.9%), complete manubriosternal (MBS) fusion (4.1%), and SXJ fusion (4.1%). Less frequently observed anomalies included: triple-ended XP (3.7%) and sternal clefts (1.5%). Rare variants, occurring in less than 1% of individuals, comprised SXPJ pseudoforamen, suprasternal bone, pseudocleft, suprasternal tubercle, and XP absence. A double-ended XP in 44% of cases was the most frequently documented variation. Other common findings included ligamentous calcification (36%), xiphoidal foramen (30%), and sternal notch (30%). The rarest anomaly was a sternal cleft, observed in only one individual (1%). These observations highlight the prevalence of minor sternal variants in the contemporary Greek population and underscore the importance of recognizing such patterns in forensic and anatomical assessments. Sternal morphological variants were distributed relatively evenly across sexes, with no significant differences observed in individual variant frequencies (Fisher’s exact test, *p* > 0.05 for all). The double-ended XP was the most common variant in both males and females. While some features, such as elongated XP, appeared more frequently in males, the differences were insignificant in this sample. These findings suggest that although sternal variants are prevalent, they do not display strong sexual dimorphism and thus may have limited utility in sex estimation. However, their presence can still aid in personal identification, particularly when multiple skeletal regions are evaluated in fragmented remains. When stratified by sex, no significant differences in variant frequency were observed (Fisher’s exact test, *p* > 0.05), supporting their role as descriptive rather than diagnostic features in sex estimation. Significantly, the distribution of specific variants differed by sex: Males more commonly exhibited suprasternal ossicles, non-fused manubrium-mesosternal joints, hyperplasia, and advanced XP development. Females more frequently presented with complete fusion of the MBSJ, aplasia and hypoplasia of the sternum, and caudal sternal clefting [42]. These findings highlight the significant variability of the sternum and underscore the importance of population-based data in understanding the frequency and distribution of these variants. Knowledge of such normal anatomical deviations is critical for radiologists, surgeons, and forensic anthropologists, helping to differentiate between normal variants and pathological conditions. While these sternal anomalies are not individually predictive of sex, their recognition contributes to the overall biological profile and may support identification when combined with traits from other skeletal regions. No significant association between SSA variants and sex was found in one study [5], contradicting earlier findings that reported a weak correlation between SSA and sex [42]. These inconsistencies suggest that SSA variations may not possess reliable predictive value for sex determination. Significant differences in the MB and SB fusion patterns between sexes were noted in another study [15], with higher fusion rates in males. In contrast, opposite results were reported elsewhere [42]. Females exhibited a higher fusion rate (19.2%) than males (5.4%). These discrepancies may reflect population-specific sexual dimorphism in SSA, emphasizing the need for further research with larger, geographically diverse cohorts and standardized assessment criteria. In terms of specific anomalies, the sternal aperture was more frequently observed in males (5.6%) than females (3.0%), whereas sternal caudal clefting was more prevalent in females (16.45%) than males (7.8%) [42]. In the French population, the SF appeared more often in males (6.5% versus 3.2% in females), with only one female exhibiting caudal clefting. However, statistical analysis in Spanish and French populations failed to demonstrate significant sex-related differences. SF is essential because vital structures like the pericardium and pleura are posterior. Due to its high incidence and large area, preliminary screening is necessary before clinical applications [59]. Additionally, SF is a significant embryological variation in forensic medicine and medical education [60]. Recent research highlights the importance of combining anatomical variants across multiple skeletal regions to enhance forensic identification, particularly in cases of fragmentary remains. For example, cranial non-metric traits—such as accessory sutures, parietal foramina, and frontal sinus patterns—have been extensively studied for their frequency and potential forensic utility [61]. Palamenghi et al. [61] emphasized that some cranial variants occur with population-level predictability and can aid in developing biological profiles. Similarly, Campobasso et al. [62] demonstrated the diagnostic value of craniofacial radiographic comparisons, especially in severely burned cases where unique structures like frontal sinuses or dental restorations remained intact. Although sternal variants (e.g., bifid XP and SF) may not exhibit strong sex associations, their inclusion alongside cranial and postcranial features could improve accuracy in sex estimation and personal identification, particularly when only partial remains are available. Sexual dimorphism was more evident in XP. One study found that females had a higher incidence of XP development [5], supported by further investigations in French populations [42]. Conversely, males in both Spanish and French populations exhibited more advanced XP development, while features such as aplasia and hypoplasia were more frequent in females. Overgrowth of the mesosternum, often accompanied by an additional SBr, was predominantly observed in males, supporting the hypothesis of hyperostotic traits in males and hypostatic characteristics in females [33]. Additionally, it has been reported that females possess fewer sternum bones than males [30]. According to Rojas et al. [42], the expression of sternal morphological variation and sex is specific to each population. Future studies need to standardize and provide valuable insights into the variability of sternal morphological characteristics. In a manner analogous to the present study that identified the influence of sex on sternum morphometrics, Toy et al. [63] conducted a research endeavor to estimate sex through machine learning algorithms, utilizing parameters gleaned from CT images of the cranium. Likewise, Mello-Gentil [64] utilized skulls and cervical vertebrae for this estimation. At the same time, Mutlu et al. [65] focused on the hyoid bone.

### 4.3. Limitations

This study has several limitations that should be acknowledged. The sample size (n = 100) was relatively small and sourced from a single geographic region (Larissa, Greece), which may limit the generalizability of the findings. Nonetheless, the morphometric evaluation was comprehensive, involving analysis of fifteen distinct parameters. Notably, while the sternum index did not yield significant results, its absolute values did reach significance. This discrepancy is likely attributable to the limited sample size. Furthermore, additional sternal measurements in the logistic regression analysis did not enhance the model’s predictive accuracy.

## 5. Conclusions

This study demonstrates that MDCT-based sternal morphometry—particularly measurements such as MBL and SBL—offers reliable indicators for sex estimation in a contemporary Greek population. Diagnostic thresholds for these parameters showed high predictive performance and may serve as practical tools in forensic anthropology when traditional skeletal markers are absent. However, the relatively small and region-specific sample (Larissa, Greece) limits broad applicability. These findings highlight the necessity of developing population-specific reference standards due to known anatomical variability across ethnic and geographic groups. Future research should aim to validate these results in larger, more diverse cohorts. Lastly, this study underscores the utility of MDCT imaging as a powerful, noninvasive modality for generating detailed 2D and 3D anatomical data, enhancing both forensic investigations and anthropological research.

## Figures and Tables

**Table 1:** The results of the morphometric measurements of the present study are distributed by sex (F—females, and M—males), SL—total sternum length, MB—manubrium, and SSA—sternal surface area.

Variables	Sex	N	Mean	Median	SD	Range	Min	Max	SE	t-Statistic	*p*
Age	F	50	58.10	63.00	17.70	61	24	85	2.50	−1.03	0.304
	M	50	61.38	60.50	13.78	60	27	87	1.95		
MB length (ΜΒL)	F	50	45.13	44.90	4.45	21.30	36.20	57.50	0.63	−5.82	<0.001
	M	50	50.50	50.75	4.79	17.70	41.30	59.00	0.68		
MB width (MΒW)	F	50	62.65	62.30	8.91	38.00	46.80	84.80	1.26	−7.10	<0.001
	M	50	78.01	77.60	12.45	67.50	51.20	118.70	1.76		
SB Length (SBL)	F	50	88.18	87.80	10.05	52.80	61.40	114.20	1.42	−9.58	<0.001
	M	50	108.13	107.45	10.75	43.40	89.60	133.00	1.52		
SB width at the 2nd intercostal space (SBWa)	F	50	25.10	24.90	3.14	16.30	16.70	33.00	0.44	−5.01	<0.001
	M	50	28.27	27.35	3.20	15.40	20.50	35.90	0.45		
SB width at the 3rd intercostal space (SBWb)	F	50	27.52	27.30	3.78	21.80	21.30	43.10	0.53	−4.72	<0.001
	M	50	31.27	31.00	4.16	15.80	23.70	39.50	0.59		
SB width at the 4th intercostal space (SBWc)	F	50	30.91	30.05	5.24	26.10	24.40	50.50	0.74	−4.34	<0.001
	M	50	35.48	35.65	5.29	23.20	24.60	47.80	0.75		
MB minimum thickness (MΒT_min_)	F	50	8.62	8.55	1.11	5.40	6.30	11.70	0.16	−4.19	<0.001
	M	50	9.61	9.90	1.26	6.20	5.90	12.10	0.18		
MB maximum thickness (MΒT_max_)	F	50	13.49	13.45	1.58	8.40	10.70	19.10	0.22	−4.78	<0.001
	M	50	15.07	14.90	1.70	7.20	11.40	18.60	0.24		
SB thickness (SBT) at the SBr 2–3	F	50	9.38	9.40	1.12	5.60	6.90	12.50	0.16	−4.28	<0.001
	M	50	10.33	10.50	1.11	4.40	8.20	12.60	0.16		
Sternum body thickness (SBT) at the SBr 4–5	F	50	8.93	8.90	1.15	5.20	6.10	11.30	0.16	−3.07	0.003
	M	50	9.68	9.70	1.27	5.70	7.10	12.80	0.18		
SL (MBL + SBL)	F	50	133.31	132.80	9.85	47.70	112.70	160.40	1.39	−12.23	<0.001
	M	50	158.63	158.55	10.84	44.30	139.50	183.80	1.53		
XP length (XPL)	F	50	35.98	34.95	13.53	51.80	12.20	64.00	1.91	−3.61	<0.001
	M	50	45.50	46.90	12.89	51.60	15.40	67.00	1.82		
XP thickness (XPT)	F	50	7.02	7.15	1.65	7.10	3.70	10.80	0.23	−3.00	0.003
	M	50	8.15	8.05	2.09	10.10	4.00	14.10	0.30		
Sternal Angle (SA)	F	50	164.04	164.50	7.36	40.00	140.00	180.00	1.04	0.81	0.420
	M	50	162.83	162.45	7.60	28.30	149.20	177.50	1.08		
Sternal Index (SI) (MBL/SBL) × 100	F	50	52.02	51.35	9.28	47.40	36.20	83.60	1.31	2.88	0.005
	M	50	47.25	47.00	7.11	28.60	33.90	62.50	1.01		
MB Index (MBI) (MBW/MBL) × 100	F	50	139.78	138.75	21.82	114.60	96.30	210.90	3.09	−3.07	0.003
	M	50	156.17	154.45	30.80	153.80	99.20	253.00	4.36		
SB index (SBI) (SBWa/SBWc) × 100	F	50	82.59	82.55	12.94	52.10	56.20	108.30	1.83	0.83	0.411
	M	50	80.68	79.70	10.09	44.50	64.00	108.50	1.43		
SSA (MB + SB) × (MBW + SBWa + SBWc)/3	F	50	5271.52	5194.10	657.65	3050.50	4082.00	7132.50	93.01	−12.12	<0.001
	M	50	7517.09	7354.35	1133.62	5221.30	5721.60	10,942.90	160.32		

Logistic Regression Analysis: The logistic regression model was statistically significant and demonstrated high predictive performance for sex classification (*p* < 0.001). Odds ratios represent the change in odds of classifying a subject as male for each one-unit increase in the predictor variable, assuming all other variables remain constant. Two morphometric predictors were retained in the final model: MBL: OR = 1.48 and SBL: OR = 1.29.

**Table 2:** Logistic regression model and performance measures, MB—manubrium, SB—sternum body.

Predictor	Estimate	SE	Z	*p*	Odds Ratio
Intercept	−43.06	9.49	−4.45	<0.001	2 × 10^−19^
MB length (mm)	0.39	0.11	3.67	<0.001	1.48
SB length (mm)	0.25	0.06	4.39	<0.001	1.29
Predictive measures	Accuracy: 0.91	Specificity: 0.90	Sensitivity: 0.92	AUC: 0.97	

**Table 3:** Sternum Metric Thresholds determined by Youden’s J, MB—manubrium, SB—sternum body, SL—total length of the sternum.

Metric	Threshold (mm)	Sensitivity (%)	Specificity (%)	PPV (%)	NPV (%)	Youden’s Index	AUC
MB length (MBL)	47.3	76	74	75	76	0.5	0.795
MB width (MBW)	70.9	78	86	85	80	0.64	0.847
SB length (SBL)	94.4	92	78	81	91	0.7	0.918
SB width at the 2nd intercostal space (SBWa)	25.6	86	64	70	82	0.5	0.772
SB width at the 3rd intercostal space (SBWb)	29.8	62	78	74	67	0.4	0.761
SB width at the 4th intercostal space (SBWc)	31.5	78	70	72	76	0.48	0.764
MB minimum thickness (MBT_min_)	9.6	64	82	78	69	0.46	0.737
MB maximum thickness (MBT_max_)	13.9	80	66	70	77	0.46	0.771
SB thickness of sternebrae 2–3	10.1	64	76	73	68	0.4	0.792
SB thickness of sternebrae 4–5	9.1	70	58	63	66	0.28	0.662
SL (MBL + SBL)	143.2	96	86	87	96	0.82	0.965
XP length (XPL)	33.1	84	50	63	76	0.34	0.687
XP thickness (XPT)	7.7	58	70	66	63	0.28	0.662
MB Index (MBI) (MBW/MBL) × 100	150.9	60	72	68	64	0.32	0.674
SSA (mm^2^) (MB + SB) × (MBW + SBWa + SBWc)/3	6039.5	96	88	89	96	0.84	0.978

**Table 4:** Frequency and Sex-Based Distribution of Sternal Morphological Variants (n = 100), XP—xiphoid process, SF—sternal foramina (xiphoid area), MB—manubrium.

Morphological Variant	Number of Cases	Frequency (%)	Male Cases (n = 50)	Female Cases (n = 50)	*p*-Value
Double-ended XP	44	44%	22	22	1.000
Ligament Calcification	36	36%	20	16	0.532
Sternal Foramen (SF)	30	30%	15	15	1.000
Sternal Notch	30	30%	17	13	0.513
Suprasternal Tubercle	19	19%	10	9	1.000
Elongated XP	14	14%	9	5	0.388
Sternal Sclerotic Band	12	12%	7	5	0.758
Suprasternal Bone	8	8%	5	3	0.715
MB Foramen	6	6%	2	4	0.678
Sternal Foramen	5	5%	2	3	1.000
Pseudoforamen	5	5%	3	2	1.000
Triple-ended XP	3	3%	2	1	1.000
Manubriosternal Fusion (MBSF)	3	3%	1	2	1.000
Sternal Cleft	1	1%	1	0	1.000

## Data Availability

All data are available to the corresponding author upon reasonable request.
